# A Journey of a Thousand Miles Begins with a Single Step

**DOI:** 10.5005/jp-journals-10071-23184

**Published:** 2019-06

**Authors:** Atul P Kulkarni

**Affiliations:** Division of Critical Care, Department of Anaesthesiology, Critical Care and Pain, Tata Memorial Hospital, Homi Bhabha National Institute, Parel, Mumbai, India

## Abstract

**How to cite this article:** Kulkarni AP. A Journey of a Thousand Miles Begins with a Single Step. Indian J Crit Care Med 2019;23(Suppl 2):S97.

“The human brain is the universe's most implausible science experiment.”

—*Will Boast, Daphne*

Today Indian Journal of Critical Care Medicine makes a new beginning. This supplement on Neurocritical Care is the first in the queue of quarterly Supplements of the Indian Journal of Critical Care Medicine. The editorial board has decided to bring out theme based special issues for the practicing as well as aspiring intensivists. In these special issues, we plan to tackle topics, which are off the beaten track, and not included in the common textbooks on Critical Care Medicine. May this series last long, and go on and on. Thanks are due to the first group of friends, who volunteered to edit this special issue on Neurocritical Care, Sameer Jog and Kapil Zirpe, both members of Editorial Board of Indian Journal of Critical Care Medicine. It was an envious task with no other volunteers, so three cheers for them!

The great poliomyelitis epidemic in Denmark, was the seed, which germinated in to the tree of Critical Care Medicine.^1^ It gave rise to the concept that all patients who were acutely sick should be cared for in a single location, which was already floating around in the USA at that time. Critical illness that time was defined as having the need to ventilate the patients.

Max Harry Weil took the concept forward in the form of shock wards where; these very sick patients were looked after by doctors doing rounds, and nurses who were experienced in the care of these patients. Usage of the term Critical Care for the first time is attributed to Max Weil. Schoemaker, a trauma surgeon and the proponent of oxygen debt in postoperative critical care; and Peter Safar, a contributor of many concepts in cardiopulmonary resuscitation; added their expertise and developed the concept of critical care further.

Dandy, a neurosurgeon is credited with the development of neurocritical care at the John Hopkins institute, nearly 90 years back. With a contribution of many giants in neurosurgery and neurology there have been many advances in neurocritical care.^2^

The subspecialties in critical care medicine keep evolving at breakneck speed, hope we can keep abreast of the advancements in our field. I wish you a happy reading.
